# Comparing complete organelle genomes of holoparasitic *Christisonia kwangtungensis* (Orabanchaceae) with its close relatives: how different are they?

**DOI:** 10.1186/s12870-022-03814-3

**Published:** 2022-09-17

**Authors:** Chi Zhang, Qianshi Lin, Jiayin Zhang, Zihao Huang, Peng Nan, Linfeng Li, Zhiping Song, Wenju Zhang, Ji Yang, Yuguo Wang

**Affiliations:** 1 Ministry of Education Key Laboratory for Biodiversity Science and Ecological Engineering, School of Life Sciences, Institute of Biodiversity Science, Fudan University, Shanghai, 200433 China; 2 Ecology and Evolutionary Biology, University of Toronto, Toronto, ON M5S 2Z9 Canada; 3 Tibet University-Fudan University Joint Laboratory for Biodiversity and Global Change, College of Science, Tibet University, Lhasa, 850012 China

**Keywords:** Christisonia, Plastid genome, Mitochondrial genome, Transcriptome, Horizontal gene transfer, Intracellular gene transfer

## Abstract

**Background:**

Orobanchaceae is the only flowering plant family with species from free-living nonparasite, hemi-parasite to holoparasite, making it an ideal system for studying the evolution of parasitism. However, both plastid and mitochondrial genome have been sequenced in only few parasitic species in Orobanchaceae. Therefore, further comparative study is wanted to investigate the impact of holoparasitism on organelle genomes evolution between close relatives. Here, we sequenced organelle genomes and transcriptome of holoparasitic *Christisonia kwangtungensis* and compared it with its closely related groups to analyze similarities and differences in adaption strategies to the holoparasitic lifestyle.

**Results:**

The plastid genome of *C. kwangtungensis* has undergone extensive pseudogenization and gene loss, but its reduction pattern is different from that of *Aeginetia indica*, the close relative of *C. kwangtungensis*. Similarly, the gene expression detected in the photosynthetic pathway of these two genera is different. In Orobanchaceae, holoparasites in Buchnereae have more plastid gene loss than Rhinantheae, which reflects their longer history of holoparasitism. Distinct from severe degradation of the plastome, protein-coding genes in the mitochondrial genome of *C. kwangtungensis* are relatively conserved. Interestingly, besides intracellularly transferred genes which are still retained in its plastid genome, we also found several horizontally transferred genes of plastid origin from diverse donors other than their current hosts in the mitochondrial genome, which probably indicate historical hosts.

**Conclusion:**

Even though *C. kwangtungensis* and *A. indica* are closely related and share severe degradation of plastome, they adapt organelle genomes to the parasitic lifestyle in different ways. The difference between their gene loss and gene expression shows they ultimately lost photosynthetic genes but through different pathways. Our study exemplifies how parasites part company after achieving holoparasitism.

**Supplementary Information:**

The online version contains supplementary material available at 10.1186/s12870-022-03814-3.

## Background

As genetic materials contained within the cell but outside the nucleus, organelle genomes of parasitic plants exhibit diverse adaptations to parasitism. Protein-coding genes in plastid genomes undergo varying degrees of degradation as parasites don’t rely on or even cannot do photosynthesis [1, 2], while those from mitochondrial genomes are rarely affected [3], with a few exceptions, e.g., the mitochondrial genome of hemi-parasitic species of Viscaceae has been severely degraded [4–6].

Previous studies have proposed models for the plastid genome evolution in parasites based on the order of gene losses [7–9]. The five stages in these models include “Photosynthetic”, “Degradation I”, “Stationary”, “Degradation II” and “Absent” stages. Genes loss started with *ndh* genes, *psa*/*psb* genes and *rpo* genes then followed by *atp*, *rbcL*, *rpl*, and *rps* genes. Recently, Liu et al*.* (2020) found a clear difference between clades when considering pseudogenization and gene loss separately in the phylogenetic framework, which indicates that exploring pseudogenization within the phylogeny will help us understand the evolutionary process of plastid genome degeneration [10]. Wicke et al. (2016) suggested that changes in parasitic lifestyle lead to the gradual loss and pseudogenization of plastid genomes [7]. Photosynthesis-associated genes are affected first, then metabolism-associated genes, and other genes at last. Different parasitic lifestyles have different degrees of gene degradation, making parasitic plants a good material for studying plastid genome evolution. The comparison of stages of parasites between different clades is beneficial for understanding the evolutionary rule of plastid genes among related species.

In addition, the fate of lost plastid genes is also an interesting topic. In holoparasitic *Orobanche*, Cusimano and Wicked (2016) found that most of lost plastid genes have been transferred into their mitochondrial genome or nuclear genome, indicating the occurrence of intracellular gene transfer (IGT) [11]. In *Cistanche*, lost plastid genes may also have been transferred into the mitochondrial genome or nuclear genome [10].

The structure of mitochondrial genome in angiosperms is complex and changeable, with low stability and high diversity of interspecific sequences [12, 13], and can have a variety of forms. It usually contains one to more circular DNA molecules, with an exception of linear mitochondrial genomes in a few plants (such as *V. scurruloideum* and *V. album*) [4, 5, 14, 15]. The gene number in the plant mitochondrial genome is highly conservative. In general, it contains about 40 genes, including core genes (e.g., *atp*, *ccm*, *cox*, *cob*, *nad*, *matR*, *mttB*) and variable genes (e.g., *rpl*, *rps*, *sdh*) [16, 17].

Unlike widely reported plastid genomes, variable structures make it difficult to obtain the complete mitochondrial genome. The difficulties of collecting materials in the field further limit the amount of mitochondrial genomes sequenced of parasitic plants. So far, only few parasites from Orobanchaceae, Balanophoraceae, Cynomoruaceae, and Viscaceae have complete mitochondrial genome data [3, 4, 18, 19].

Orobanchaceae is the largest parasitic family in angiosperms, which contains different lifestyles, from autotroph, hemi-parasitsm to holoparasitsm. The well-studied lifestyle and phylogenetic relationships among species of Orobanchaceae provide us an excellent system to study dynamic changes of components of organelle genome, the fate of lost genes, and horizontal gene transfer (HGT) during holoparasitic evolution.

Previous phylogenetic studies of Orobanchaceae [20, 21] confirmed that the family is composed of six main clades: Clade I, *Lindenbergia*; Clade II, Cymbarieae; Clade III, Orobancheae; Clade IV, Pedicularideae; Clade V, Rhinantheae, and Clade VI, Buchnereae. Among them, Buchnereae and Rhinantheae contain both hemi-parasites and holoparasites [21].

At present, plastid genomes of more than 20 genera of Orobanchaceae have been reported. These reports indicate that the genome of non-parasites, such as *Lindenbergia*, does not undergo any plastid genome degradation [1, 22]. However, plastid genes of holoparasitic genera, such as *Aeginetia indica*, have undergone gene loss and pseudogenization [23].

In Orobanchaceae, only *Castilleja paramensis* and *A. indica* have complete genome data of both plastid and mitochondrion [3, 23, 24]. Previous studies showed that the plastid genome of *A. indica* has undergone severe degradation. Massive genes, especially photosynthetic-associated genes have been lost or pseudogenized. In addition, several IGT events of plastid loss genes and HGT events from known hosts have also been found in the mitochondrial genome of *A. indica* [23]. However, whether these phenomena exhibit a clade specificity, or represent a general rule across different clades remains to be studied.

*Christisonia* is sister to *Aeginetia.* According to phylogenetic analyses, *Christisonia* and *Aeginetia*, along with *Hyobanche*, *Harveya*, form a monophyletic holoparasitic lineage in Buchnereae [20, 25]. The species of *Christisonia* usually parasitize on roots of Bambusoideae (Poaceae) [26].

In this study, we sampled *Christisonia kwangtungensis* in the Danxia area, Guangdong, China [27]. We sequenced and assembled the complete plastid genome and mitochondrial genome of this species. By comparing the published organellar genomic data from the same clade and other clades in Orobanchaceae, we attempted to characterize organellar similarities/differences among Buchnereae and across different clades. Based on the transcriptomic data of *C. kwangtungensis*, we explored the effect of parasitic life on genes related to photosynthesis. Our results could help to find out the effect of parasitic life on the organelle genome of parasitic plants, elucidate the coevolutionary relationship of plastid genome and mitochondrial genomes, and understand similarities/differences among different parasites in Orobanchaceae.

## Results

### Structure and characteristics of the plastid genome of *C. kwangtungensis*

The plastid genome of *C. kwangtungensis* is 96,074 bp in length and consists of a long single-copy (LSC) region (30,072 bp), a small single-copy (SSC) region (480 bp), and two inverted repeat (IR) regions (32,761 bp for each). The total length is much smaller than that of most photosynthetic angiosperms. GC content of this plastid genome is 34.7% (Figure S1). We annotated 68 intact genes and 11 pseudogenes in the plastid genome of *C. kwangtungensis.* Among them, intact genes include 31 tRNA genes, 8 rRNA genes, 10 *rps* genes, 7 *rpl* genes, and 12 other genes (*ycf1*, *ycf2*, *accD*, *matK*, *infA*, *clpP*, *psbA*, *atpA*, *atpB, atpF*, *atpH*, and *atpI*). These pseudogenes are *rps16*, *psbI*, *rpoB*, *psbD*, *atpE*, *petB*, *rpoA*, *rpl23*, *ycf15*, *ndhB*, and *rps15.*

Similar to *A. indica*, the SSC region in the plastid genome of *C. kwangtungensis* is severely reduced, possessing only a *rpl32* gene. Genes located in the LSC region of most photosynthetic angiosperms (such as *rpl14*, *rpl16*, *rps3*, *rpl22*, and *rps19*) appear in the IR region of *C. kwangtungensis*. In *C. kwangtungensis*, there are also two intact *ycf1* genes in each of two IR regions, whereas one intact *ycf1* gene usually spans the IR and SSC regions in most photosynthetic angiosperms [28, 29].

Comparing to plastid genomes of photosynthetic *Lindenbergia philippehsis* and hemi-parasitic *S. asistica,* that of *C. kwangtungensis* experiences serious gene loss (Figure S2). All *psa* genes and most *psb* genes (*psbB*, *psbC*, *psbE*, *psbF*, *psbH*, *psbJ*, *psbK*, *psbL*, *psbM*, *psbN*, *psbT*, and *psbZ*) participated in photosystem I and photosystem II have lost, so do ten *ndh* genes (*ndhA*, *ndhC*, *ndhD*, *ndhE*, *ndhF*, *ndhG*, *ndhH*, *ndhI*, *ndhJ*, and *ndhK*) encoding subunits of NADH-dehydrogenase complex. Also, five *pet* genes (*petA*, *petD*, *petG*, *petL*, and *petN*) which encode cytochrome b6/f complex subunits with function in photosynthetic electron transport have lost. Besides, *ycf3* and *ycf4* (photosystem assembly factors), *ccsA* (heme attachment factor), *cemA* (encoding envelop membrane protein), *rbcL* (the large subunit of Rubisco), and two genes encoding DNA dependent RNA polymerase, *rpoC1* and *rpoC2*, have lost, while *atpE* encoding F-type ATPase subunits has become pseudogene. In holoparasitic *C. kwangtungensis* and *A. indica*, compared with hemi-parasitic *S. asiatica* (one gene lost, five pseudogenes), the number of lost genes is 4–4.6 times as many as that of *S. asiatica*, and the number of pseudogenes is 2.5–3 times that of *S. asiatica*. Whereas in Rhinantheae, compared with hemi-parasitic *M. koreanum* (seven gene lost, four pseudogenes), the number of lost genes in holoparastic *L. squamaria* is 71.4% of *M. koreanum*, and the number of pseudogenes is 7.5 times that of *M. koreanum*.

### Structural characteristics and gene transfer of mitochondrial genome of *C. kwangtungensis*

The mitochondrial genome of *C. kwangtungensis* consists of three closed-loop chromosomes with lengths of 87,933 bp, 303,254 bp, and 241,909 bp (with a length of 633,096 bp in total; Figure S3), with GC contents of 44.2%, 44.6%, and 44.8%, respectively. There are in total 75 local genes on the mitochondrial genome of *C. kwangtungensis*, including 39 protein-coding genes, 26 transporter RNA, and ten ribosomal RNA. Mitochondrial protein-coding genes distribute on all three chromosomes. The first chromosome has four local protein-coding, three transporter RNA, and two ribosomal RNA genes; the second chromosome has 16 local protein-coding, seven transporter RNA and five ribosomal RNA genes; the third chromosome has 19 local protein-coding, 16 transporter RNA and three ribosomal RNA genes. All 24 core genes (*atp*, *ccm*, *cob*, *cox*, *nad* genes, *matR*, and *mttB*) are not lost. Only several variable genes (*rps2*, *rps11*, and *rps19*) are absent.

**Fig. 1:**
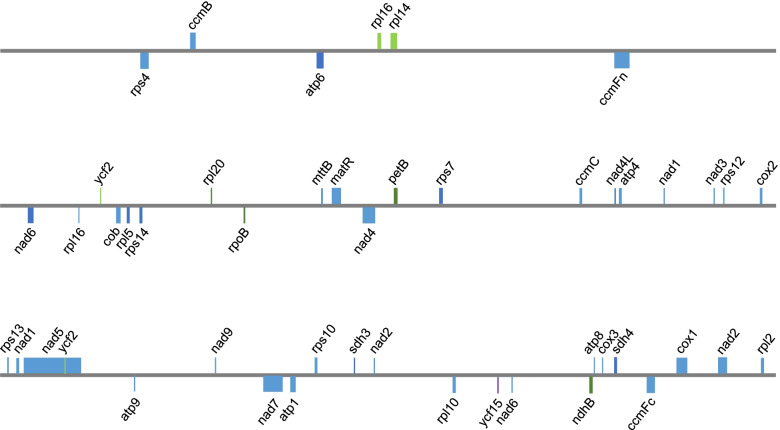
Gene distribution in mitochondrial genome of *C. kwangtungensis.* Squares with different colors represent genes from different sources (blue, mitochondrial local genes; brown, mitochondrial genes of unknown origin; light green, exogenous HGT plastid genes; dark green, plastid gene fragments from intracellular transfer; purple, plastid gene fragments from unknown sources)

**Fig. 2:**
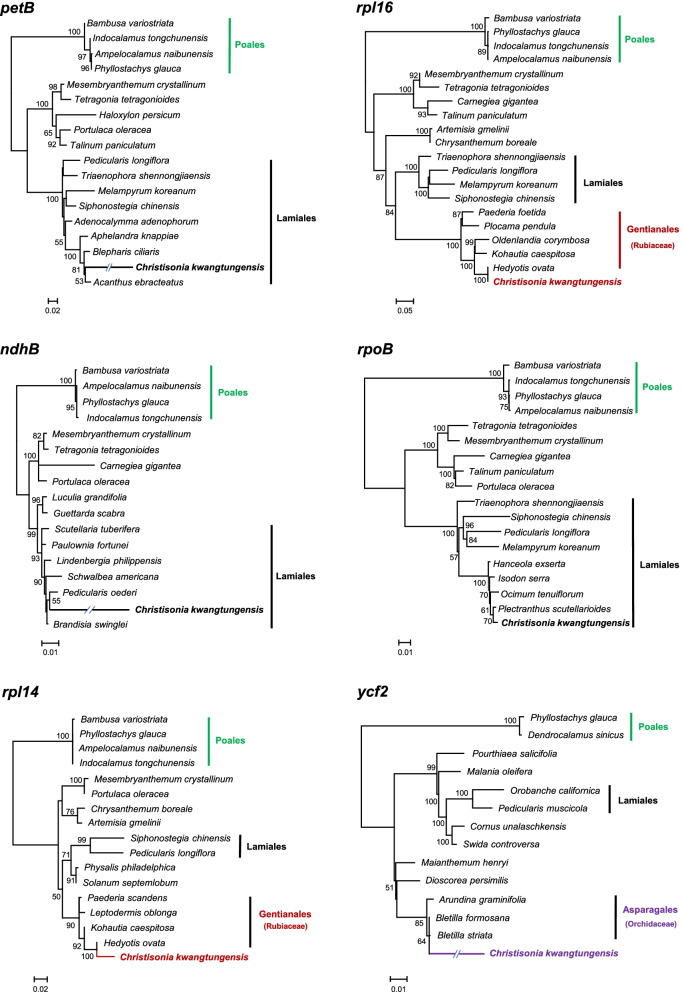
Maximum likelihood phylogeny of plastid-derived genes in the mitochondrial genome of *C. kwangtungensis*. Phylogenetic trees show evidence of horizontal gene transfer and intracellular gene transfer. The clade length of trees represents the base substitution rate, clades which are too long are shortened by "/ /". Only bootstrap values >50% are shown

In addition to local mitochondrial protein-coding genes, there are also several plastid-derived gene fragments in the mitochondrial genome of *C. kwangtungensis* (Fig. 1). These fragments are probably unable to be translated because their ORFs are incomplete. A total of nine plastid-derived transferred gene fragments of eight genes (two *ycf2* fragments) are found in the mitochondrial genome. Interestingly, homologs of these eight genes are still present in the plastid genome of *C. kwangtungensis*. Phylogenetic analyses performed on all plastid transferred fragments (Fig. 2) show that four fragments (*rpoB*, *ndhB*, *rpl20*, and *petB*) were derived from intracellular plastid gene transfer, three fragments (*ycf2*-1, *rpl14*, and plastid *rpl16*) were transferred from species other than the members of Lamiales (Table S1), and two fragments are too short to determine their donor (*ycf15* and *ycf2*-2).

Among three horizontally transferred plastid genes from other species, we did not find any putative donor from Poaceae, the current host lineage of *C. kwangtungensis*. Phylogenetic evidence supports that the *ycf2* gene fragment might be transferred from Orchidaceae (85% BS; sister to *Bletilla*, 64% BS), while *rpl16* and *rpl14* fragments were transferred from the Gentianaceae (100% BS).

### The degeneration of the photosynthesis pathway in *C. kwangtungensis* revealed by transcriptome analysis

We obtained a total of 6.67 Gb clean transcriptomic reads. After assembly and redundancy removal, we obtained a total of 150,489 transcripts and extracted 83,525 as unigenes. The total length, average length, N50, and GC contents were 80,696,667 bp, 966 bp, 1,672 bp, and 43.51%, respectively. By comparing unigenes to seven functional databases, there are 48,560 (NR: 58.14%), 42,692 (NT: 51.11%), 29,942 (Swissprot: 35.85%), 16,846 (COG:20.17%), 35,805 (KEGG: 42.87%), 11,934 (GO: 14.29%), and 31,025 (Interpro: 37.14%) unigenes were annotated. According to annotation results, a total of 47,710 CDS were detected and got 2,899 CDS by ESTScan forecasting in unigenes which were not annotated. At the same time, 9,276 SSRs were detected in 7,749 unigenes, and 1,405 unigenes encoding transcription factors were predicted.

**Fig. 3:**
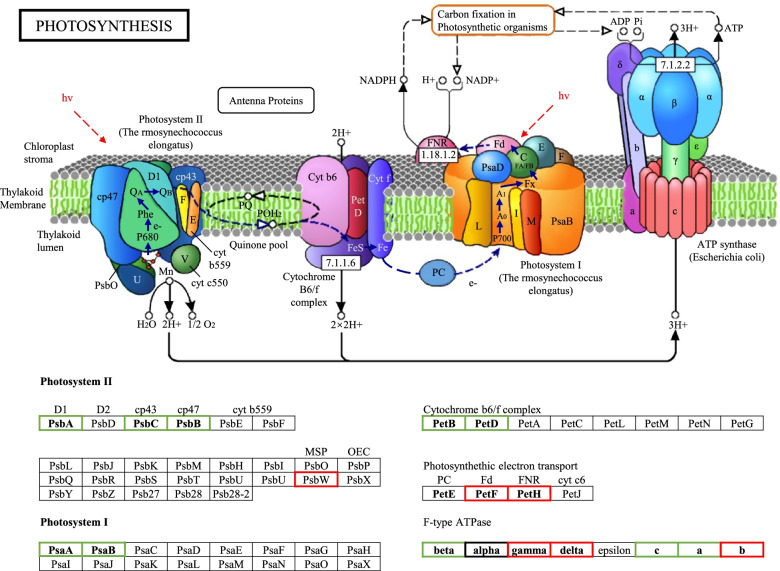
Gene expression in the photosynthesis pathway observed in the *C. kwangtungensis* and *A. indica* transcriptome. Genes with detected expression in *C. kwangtungensis* are in green boxes. Genes with detected expression in the transcriptome of *A. indica* are in red boxes [23]. Genes with detected expression in both species are in black box. With courtesy of © www.genome.jp/kegg/kegg1.html

According to the KEGG pathway database, the photosynthesis pathway (ko00195) in plants consists of 63 genes (30 plastid genes and 33 nuclear genes). In the plastid genome of *C. kwangtungensis*, all genes encoding PS I and PS II except *psbA* have been lost or undergone pseudogenization. Also, all genes of cytochrome *b6f* complex and photosynthetic electron transport have lost or became pseudogenes. Most of the Ftype ATPase-related genes are intact, and only *atpE* has been pseudogenized. Based on transcriptome analysis, only eleven photosynthetic pathway genes were detected. These genes include three genes encoding PS II, two genes encoding PS I, two genes encoding proteins involved in photosynthetic electron transport, and four genes encoding F-type ATPase components (Fig. 3). No expression of other genes in this pathway was detected, indicating that these genes have been lost or are not expressed. Though plastid and transcriptome analyses, it can be shown that *C. kwangtungensis* has completely lost its photosynthetic pathway.

The porphyrin and chlorophyll metabolism pathway (ko00860) from the KEGG pathway database contains many complex processes. We detected the expression of nine genes (*HemA*, *HemL*, *HemB*, *HemC*, *HemD*, *HemE*, *HemF*, *HemN*, and *HemY*) in the pathway from glutamate to protoporphyrin IX in the transcriptome of *C. kwangtungensis* (Figure S6). However, due to the absence of expression of divinyl chlorophyllide an 8-vinyl-reductase [EC:1.3.1.75], which catalyzes divinyl protochlorophyllide to protochlorophyllide, the chlorophyll synthesis pathway appears to end at divinyl-proto-chlorophyllide production in *C. kwangtungensis*. The chlorophyll synthesis pathway is obviously incomplete in the later stage, thus *C. kwangtungensis* could not synthesize chlorophyll.

## Discussion

### Species of Orobanchaceae experience different degrees of plastid genome reduction

The degeneration of plastid genome between different clades of Orobanchaceae exhibits significant difference, which may be related to the timing of transformation to their parasitic lifestyle. In Orobanchaceae, there are two clades (Rhinantheae, Buchnereae) that contain both hemi-parasitic and holoparasitic species [20, 21]. The plastomic size of hemi-parasites and holoparasites in Buchnereae is significantly different: The plastomic size of *C. kwangtungensis* (96,074 bp) is similar to another holoparasitic species *A. indica* (86,212 bp) [23], but much smaller than their hemi-parasitic relative *S. asistica* (184,887 bp, BK016276). In Rhinantheae, the plastid genome of holoparasitic *Lathraea squamaria* (150,504 bp, KM652488) is similar in size to hemi-parasitic *Melampyrum koreanum* (143,865 bp, MW463054) and *Euphrasia regelii* (153,026 bp, MK070895) [11], indicating that these two clades have experienced different degrees of plastid degradation (gene loss and pseudogenization) during the evolution from hemi-parasitism to holoparasitism.

**Fig. 4:**
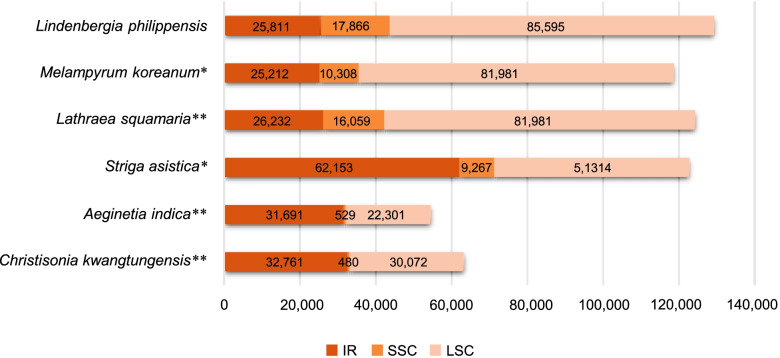
Comparison of plastome structure of representative Orobanchaceae species with different lifestyles. The length of SSC region (orange), LSC region (pink), and IR region (brown) are shown in the bar plot. Parasitic types are noted by * (with no *, non-parasitic; *, hemi-parasitic; **, holoparasitic)

As to the structure of plastid genomes, there are also extensive differences between Buchnereae and other clades. Compared to the non-parasitic *L. philippehsis*, the SSC region of hemi-parasitic *M*. *koreanum* in Rhinantheae has a 42.4% reduction in size, but no significant reduction in other regions (Fig. 4). However, the IR region of *S. asistica* in Buchnereae has expanded by 164%, whereas the LSC and SSC regions have decreased by 40.0% and 48.2%, respectively.

During the transition from hemi-parasitism to holoparasitism, compared with *M. koreanum*, *L. squamaria* has a 55.8% expansion in the SSC region, but no significant change in the LSC region and IR region. Compared with *S. asistica*, in holoparasitic *C. kwangtungensis* and *A. indica*, IR region is shortened by 47.3%-49.0%, LSC region is shrunk by 41.4%-56.5%, and SSC region is reduced more severely, reaching 94.3%-94.8%. Previous studies have demonstrated that the expansion and reduction of the SSC region is one of the main reasons for structural changes of plant plastid genomes [24]. In this study, the plastid genome structure of Rhinantheae shows a clade specificity that differs from other clades during the transition of lifestyle.

**Fig. 5:**
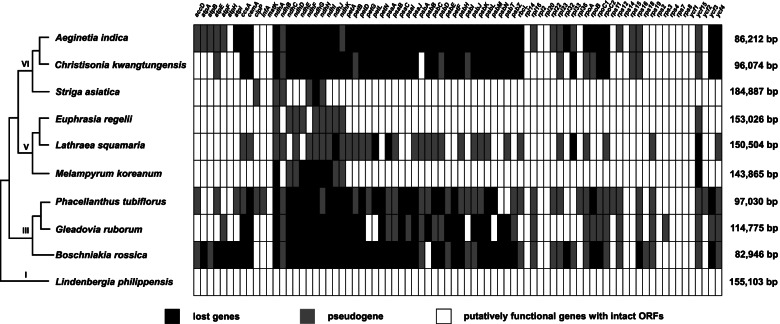
Plastome gene content of ten Orobancheae species including *A. indica* and *C. kwangtungensis*. The tree topology on the left is a schematic diagram drawn according to Maximum Likelihood phylogenetic tree constructed with plastid genes (Figure S8) and phylogenetic analyses in Fu et al*.* [21]. The color of box represents physically lost genes (black), pseudogenized genes (grey), or putatively functional genes with intact ORFs (white)

After the establishment of holoparasitic lifestyle, all nutrients, inorganic salts, and water required by the non-photosynthetic plant are obtained from its host. Holoparasites have lost the capability of photosynthesis, thus photosynthesis-associated genes on plastid genomes will be degraded to different degrees [1, 10, 30]. Based on models of plastid genome evolution in parasites proposed by previous studies [7–9, 28], *C. kwangtungensis* is similar to *A. indica*, which is in the “Stationary” stage, whereas the plastid genome of *L. squamaria* is in the “Degradation I” stage. Compared with *A. indica* (46 genes were lost, 15 genes became pseudogenes), both loss and pseudogenization of genes in *C. kwangtungensis* are a little less (40 genes were lost and 11 genes became pseudogenes) (Fig. 5). Some genes in the state of loss and pseudogene in *C. kwangtungensis* and *A. indica*, can provide clear evidence that gene loss may undergo pseudogenization preceding to complete loss of genes [10]. Compared with Rhinantheae, Buchnereae has more severe gene loss during the transformation from hemi-parasitism to holoparasitism.

As the only holoparasite of Rhinantheae, *L. squamaria* undergone relatively less gene loss in its plastid genome. There is also not much different in plastid genome between *L. squamaria* and hemi-parasitic plants of the same clade, indicating that *L. squamaria* maybe just in an early stage of holoparasitism. However, in Buchnereae, plastid genomes of *C. kwangtungensis* and *A. indica* have massive gene loss. These two species are nested deep in a clade of holoparasitic species, suggesting that they may be at a later stage of holoparasitism, which makes their plastid genomes significantly different from those of hemi-parasitic species of the same clade. Considering there are much more holoparasitic species in Buchnereae than Rhinantheae, the more drastic change in plastid genomes of Buchnereae may reflect its longer history of holoparasitism. Models of plastid genome evolution in parasites also confirm this conclusion.

### The influence of parasitic lifestyle on gene loss in mitochondrial genome

Although the parasitic lifestyle has a significant impact on the gene content and structure of plastid genome, it is usually not expected to affect mitochondrial genes, especially core genes. Except for the massive gene loss of hemi-parasitic species of Viscaceae, mitochondrial genomes of most parasites are not highly degraded [3, 18]. The mitochondrial genome of *C. kwangtungensis* is also consistent with this rule. Its core genes do not experience gene loss or pseudogenization. Several variable genes (two ribosomal protein subunit genes, *rps2* and *rps11*; two respiratory genes, *sdh3* and *sdh4*) have been lost. In *A. indica,* besides these gene loss events listed above, it has lost two other ribosomal protein subunit genes, *rps1* and *rps7* [24]. Since mitochondrial genes mentioned above also have loss events in autotrophic plants, those gene losses show no clade specificity or parasitism specificity [3, 13, 24, 31, 32] (Figure S7). Our results support that mitochondrial core gene content is not under the influence of parasitic lifestyle.

### Differences in IGT and HGT between *Christisonia* and *Aeginetia*

In a previous study, Chen et al. (2020) indicated that some genes lost in the plastid genome might be transferred into mitochondrial genome in *A. indica* [23]*.* Afterwards, Choi and Park (2021) assembled the mitochondrial genome of *A. indica* and confirmed such IGT events [24]. These mitochondrial plastid insertion (mipt) are mostly fragmental, meaning that they may have lost their function. In this study, despite different methods used, we failed to detect the transfer of genes lost in plastid genome into the mitochondrial genome, which have been frequently found in many parasitic species including the sister group *Aeginetia*, and other parasitic lineages (e.g., *Pilostyles* (Apodanthaceae), and *Orobanche* (Orobanchaceae) [11, 23, 33]

The mitochondrial genome of parasitic plants is prone to accept transferred plastid genes [34]. In this study, there are eight plastid-derived gene fragments from other species found in the mitochondrial genome of *C. kwangtungensis*. All these fragments are incomplete and have lost their function. Among these plastid genes, five mipt fragments are nested within Lamiales. However, these plastid genes are not lost in the plastid genome of *C. kwangtungensis*. They may be transferred directly from the native plastid genome.

In addition, we found some genes nested within relatives of Orobanchaceae. It should be noted that some fragments are too short to carry sufficient informative sites for tracing their donors. Mutations at key sites may cause genetic deviations in inferring the origin of transferred gene [35]. Therefore, for certain transferred gene fragments, a phylogenetic topology that a parasite nested into another family shouldn’t be simply judged as the evidence of unknown host-parasite association. For example, in this study, the transferred *rpl20* gene and the informative sites of the Lamiaceae group were screened (Table S2). According to the comparison of information sites, there are not enough informative characters for constructing a confident phylogenetic tree. The A-G mutation of a key site (the 187^th^ nucleoside acid base) is responsible for nesting *C. kwangtungensis* into Lamiaceae, the closely related group of Orobanchaceae. Mutation at key informative sites of a gene suffers from informative paucity. The mutation of a single site may lead to deviations in donor judgment. In this case, Lamiaceae should not be judged as historical host of *C. kwangtungensis*.

We identified *ycf2*, *rpl14* and *rpl16* as horizontally transferred genes. In the* atpI* phylogeny, *A. indicate* was nested into monocot species which are known as its hosts [24]. However, no transferred gene from current known host of Poaceae has been detected in the mitogenome of *C. kwangtungensis*, suggesting that all these HGT genes probably come from other independent HGT events. A previous study showed HGTs in *Sapria himalayana* (Rafflesiaceae) from Cucurbitaceae and Apiaceae, indicating possible historical hosts other than the present host (grape family) [36]. In our study, the phylogenetic analysis based on *rpl16* and *rpl14* indicated that *C. kwangtungensis* might parasitize on Gentianales in the past.

### Similarities and differences in loss patterns of photosynthetic pathways

As a holoparasite, *C. kwangtungensis* does not photosynthesize [27] and obtains nutrients from its host plants entirely. The loss of photosynthetic pathway is demonstrated by the loss of photosynthesis-associated genes in the plastid genome and the undetectable expression of many photosynthetic pathway genes in the transcriptome of *C. kwangtungensis*. In the transcriptome, only the expression of *psbA*, *psbC*, *psbB*, *psaA*, *psaB*, *petB*, *petD*, and *beta*, *alpha*, *C*, *B* of F-type ATPase can be detected, which is quite different from the expression genes detected in *A. indica* (Fig. 3). The expressions detected in the transcriptome of *A. indica* are in photosynthetic electron transport, F-type ATPase and a *psbW* of PS II, with no expressions of photosystem and Cytochrome b6/f complex. However, the genes detected to be expressed are concentrated in F-type ATPase, PS I, Cytochrome *b6/f* complex, and PS II. Expression of photosynthetic electron transport cannot be detected at all. This indicates that although both *A. indica* and *C. kwangtungensis* do not have photosynthetic activity, the degradation strategies of the photosynthesis-associated genes are different. We also compared expression patterns of photosynthetic pathway in other species (Figure S5), and found apparent differences between different parasitic lineages, even among species with relatively close relationships, suggesting that the loss of photosynthetic pathway genes can be different after entering the parasitic life. The loss of photosynthetic function and the retention of some gene expression in the photosynthetic pathway mean that these gene expressions may have other function besides photosynthesis, but needs to be further tested.

Similar to *A. indica*, the expression of some key genes in porphyrin and chlorophyll metabolic pathways of *C. kwangtungensis* is not detected in the later stage, resulting in its inability to synthesize chlorophyll. Chlorophyll is the main dye that absorbs light energy during photosynthesis, and degradation of the chlorophyll synthesis pathway may be conducive to survival and evolution. As far as degradation strategies are concerned, *A. indica* and *C. kwangtungensis* share some similarities.

## Conclusion

In this study, we sequenced and assembled organelle genomes and transcriptome of *Christisonia kwangtungensis*, a holoparasite from Buchnereae of Orobanchaceae. By comparing with its closely relatives, we found that although *Christisonia* and *Aeginetia* are holoparasitic sister groups and share severe degradation of plastid genome, they still adapted organelle genomes to the holoparasitic lifestyle in different ways. The difference between gene loss and gene expression shows they ultimately lost photosynthesis but through different pathways. In Orobanchaceae, holoparasites in Buchnereae have more plastid gene loss than Rhinantheae, reflecting the relatively longer history of holoparasitism in the former lineage. Unlike *Aeginetia,* we found no gene lost in plastid genome transferred into the mitochondrial genome of *Christisonia.* Some mitochondrial HGTs are from plant lineages other than its current hosts, which indicates possible historical hosts. Therefore, future studies could pay more attention to the formation mechanisms of such organelle genome differentiation across diverse holoparasitic lineages.

## Methods

### Plant material collection, DNA extraction, and next generation whole genome sequencing

Tissues of *C. kwangtungensis* used in this study were collected from Danxia Mountain, Guangdong, China. Fresh materials used for DNA extraction were stored in absorbent silica gel at 4℃. Voucher specimens are stored in the herbarium of Fudan University (FUS). For genomic DNA extraction, we chose the top inflorescence and pedicel far away from the root to avoid plant tissues from host near the haustorium. The Plant Genomic DNA Kit (TIANGEN BIOTECH (BEIJING) CO., LTD.) was used to extract the DNA following the protocol.

The total DNA meeting the requirements of next generation sequencing was sent to BioMarker Technologies for whole genome sequencing. The sample sequencing platform was Illumina Hiseq X Ten. The size of the inset library was 350 bp, the reading length was 150 bp, and the amount of sequencing data was 20 Gb. All plastid reads were extracted from the whole genome sequencing data.

### Assembling and annotation of plastid and mitochondrial genome

The plastid and mitochondrial genome of *C. kwangtungensis* was assembled using GetOrganelle v1.7.5 [37]. Then Bandage v0.8.1 was used for manual correction [38]. The final assembly was polished using Pilon v1.22 [39]. The complete genomic sequence of both organellar genomes obtained by assembly was annotated by DOGMA (http://dogma.ccbb.utexas.edu/) [40] and GeSeq (https://chlorobox.mpimp-golf.mpg.de/geseq.html) [41]. The threshold was adjusted several times to avoid missing annotated genes. ORFfinder was used to determine the start and end positions of each gene annotated on both organellar genomes (www.ncbi.nlm.nih.gov/orffinder/). For genes that could not be determined by ORFfinder, the start and end positions of the same genes in relative species were used as references. For those putative lost genes, we mapped reads against the homologous sequences of these genes to determine whether they are truly lost. Referring to previous research methods [1, 10, 33], we defined pseudogenes as incomplete genes: abnormally short genes; or genes whose translation is terminated in advance; or genes which are significant difference from referential genes.

After the artificial curation, the final annotation file of both organellar genomes were prepared into annotation files in the format using Sequin v15.10 (www.ncbi.nlm.nih.gov/Sequin/index.html) for drawing both organellar genomes maps. Both organellar genomes maps were drawn using the online tool OGDRAW (https://chlorobox.mpimp-golm.mpg.de/OGDraw.html) [42], all the parameters selected during operation were defaulted.

### Identification of exogenous transferred fragments of mitochondrial genome

Due to the high diversity of mitochondrial genome sequences and the lack of transcriptome data of Orobanchaceae parasitic species, we only consider and discuss plastid genes or fragments derived from external sources on the mitochondrial genome of parasitic plants. Phylogenetic inconsistency is the main method for identifying horizontally transferred genes. The software BlastN v2.2.23 was used to find exogenously transferred sequences on the mitochondrial genome (e-value < 1E-10). The plastid genome sequence of non-parasite *L*. *philippehsis* was used as reference sequence for searching plastid transferred fragments, while the mitochondrial genome data of hemi-parasite *C. paramensis* and other parasitic species (Table S3) were used as reference sequences for searching mitochondrial transferred sequences.

All found exogenous transferred fragments need to be subjected to phylogenetic analysis to determine whether the source of gene fragment is intracellular plastid genome transfer (i.e., IGT) or organelle genome transfer from a putative exogenous host (i.e., HGT). The DNA sequence matrixes of genes were combined and aligned by nucleotide with MEGA v11.0.8 [43] build-in muscle [44] and adjusted manually. All missing data were replaced with gaps. Maximum likelihood (ML) phylogenetic trees were constructed using RAxML v7.0.4 [45] with the GTRGAMMA model and 1,000 bootstrap replications. If a plastid fragment in the mitochondrial genome is nested within Lamiales or Orobanchaceae, and the Lamiales or Orobanchaceae group forms a clade in the phylogenetic tree (BS > 70%), then the fragment is identified as an intracellularly transferred sequence. If the Lamiales is monotypic but the putative transferred fragment nested into non-Lamiales groups (BS > 70%), then the gene fragment is judged as a putative horizontally transferred sequence.

### Plastid phylogeny

We have performed the phylogenetic analysis based on concatenated sequences of all coding genes present in the plastid genome of *L. philippensis.* Plastid sequence data of Orobanchaceae except *C. kwangtungensis* and other families were obtained from NCBI databases (Table S4). The alignment was made by using MEGA v11.0.8 [43] build-in muscle [44] and adjusted manually. All lost genes were treated as missing data. Maximum Likelihood (ML) phylogenetic analysis was run in RAxML v 7.0.4 [45]. Support values for branches of the ML trees were assessed based on 1,000 bootstrap replicates.

### Transcriptome analysis

In order to avoid the contamination from hosts, we sequenced transcriptome of the entire plant excluding the haustorium. Raw sequencing data were filtered by removing adaptors and the data quality assessment. Transcripts were assembled through Trinity v2.12.0, then unigenes were extracted from these transcripts using a perl script in Trinity [46]. At the same time, the expression level of all unigenes was determined. Within KEGG (https://www.genome.jp/kegg), we only focused on the photosynthesis pathway (ko00195) and the porphyrin and chlorophyll metabolism pathway (ko00860) [47]. All homologs of the photosynthetic pathway were BLAST against unigenes to detect the expression of photosynthetic-associated genes in *C. kwangtungensis*.

## Supplementary Information

**Additional file 1:** **Figure S1.** The plastid genome map of holoparasitic *C. kwangtungensis*. Genes labelled outside the outer circle are transcribed clockwise, while those inside are transcribed counterclockwise. Dashed area in the inner circle indicates the GC content of plastid genome. **Figure S2.** Comparison of plastid genome structure among non-parasite (*L. philippehsis*), hemi-parasite (*S. asistica*), and holoparasite (*C. kwangtungensis*). Green lines connect homologous plastid coding genes. Green dotted lines indicate pseudogenized regions. Regions without connecting lines indicate gene loss. **Figure S3.** The mitochondrial gene map of holoparasitic *C. kwangtungensis*. Genes labelled outside the outer circle are transcribed clockwise, while those inside are transcribed counterclockwise. Dashed area in the inner circle indicates the GC content of mitochondrial genome. **Figure S4.** Maximum likelihood phylogeny of rpl20 in the mitochondrial genome of *C. kwangtungensis*. The phylogenetic tree shows evidence of intracellular gene transfer. The clade length of trees represents the base substitution rate. **Figure S5.** Gene expression in the photosynthesis pathway observed in transcriptomes of ten species. Detected expressed genes are marked as green. Species in the same clade of *C. kwangtungensis* are in the red box. With courtesy of © www.genome.jp/kegg/kegg1.html. **Figure S6.** Gene expression in porphyrin and chlorophyll metabolism pathway observed in the transcriptome of *C. kwangtungensis*. Genes with detected expression are in the red boxes. The name and number of gene expression products were marked at each node. With courtesy of © www.genome.jp/kegg/kegg1.html. **Figure S7.** Mitochondrial gene content (core genes in blue and variable genes in yellow) of 21 species including *C. kwangtungensis* and *A. indica*. Retained genes are marked in white and lost genes are marked in black. **Figure S8.** Maximum likelihood phylogenetic tree of Orobanchaceae based on concatenated sequences of all coding genes present in the plastid genome of *Lindenbergia philippensis*. **Table S1.** Statistics of putative plastid transferred genes in mitochondrial genome of *C. kwangtungensis*. Potential donors, transfer types, fragment lengths, and bootstrap of all the putative plastid transferred genes on mitochondrial gnome of *C. kwangtungensis*. **Table S2.** Comparison of informative characters of the horizontally transferred fragment *rpl20* of *C. kwangtungensis* and its related species. **Table S3.** Mitochondrial genome sequence of Lamiales used in this study. **Table S4.** Plastid genome sequence for plastid phylogeny used in this study.

## Data Availability

The raw whole-genome sequencing (WGS) and RNASeq sequence reads data of *C. kwangtungensis* were deposited in NCBI Sequence Read Archive under accession number PRJNA792417: the accession number of plant sample RNA-Seq from *C. kwangtungensis* was SAMN24424670; the accession number of plant sample WGS from *C. kwangtungensis* was SAMN24424669; the annotated plastid and mitochondrial genome sequences of *C. kwangtungensis* were deposited in GenBank under accession numbers OL362208 and OM219025-219,027, respectively. The data sets supporting the results of this study are included in this manuscript and its additional files.

## Abbreviations

IGT: Intracellular gene transfer
HGT: Horizontal gene transfer
LSC: Large single-copy region
SSC: Small single-copy region
IR: Inverted repeat
mipt: Mitochondrial plastid insertion
