# Supplementary material for: Comparing complete organelle genomes of holoparasitic Christisonia kwangtungensis (Orabanchaceae) with its close relatives: how different are they?
Source: BMC Plant Biol. 2022 Sep 17;22:444. doi: 10.1186/s12870-022-03814-3 (PMC9482287; doi:10.1186/s12870-022-03814-3)
Supplement: Supplementary file 1 — Additional file 1: Figure S1. The plastid genome map of holoparasitic C. kwangtungensis. Genes labelled outside the outer circle are transcribed clockwise, while those inside are transcribed counterclockwise. Dashed area in the inner circle indicates the GC content of plastid genome. Figure S2. Comparison of plastid genome structure among non-parasite (L. philippehsis), hemi-parasite (S. asistica), and holoparasite (C. kwangtungensis). Green lines connect homologous plastid coding genes. Green dotted lines indicate pseudogenized regions. Regions without connecting lines indicate gene loss. Figure S3. The mitochondrial gene map of holoparasitic C. kwangtungensis. Genes labelled outside the outer circle are transcribed clockwise, while those inside are transcribed counterclockwise. Dashed area in the inner circle indicates the GC content of mitochondrial genome. Figure S4. Maximum likelihood phylogeny of rpl20 in the mitochondrial genome of C. kwangtungensis. The phylogenetic tree shows evidence of intracellular gene transfer. The clade length of trees represents the base substitution rate. Figure S5. Gene expression in the photosynthesis pathway observed in transcriptomes of ten species. Detected expressed genes are marked as green. Species in the same clade of C. kwangtungensis are in the red box. With courtesy of © www.genome.jp/kegg/kegg1.html. Figure S6. Gene expression in porphyrin and chlorophyll metabolism pathway observed in the transcriptome of C. kwangtungensis. Genes with detected expression are in the red boxes. The name and number of gene expression products were marked at each node. With courtesy of © www.genome.jp/kegg/kegg1.html. Figure S7. Mitochondrial gene content (core genes in blue and variable genes in yellow) of 21 species including C. kwangtungensis and A. indica. Retained genes are marked in white and lost genes are marked in black. Figure S8. Maximum likelihood phylogenetic tree of Orobanchaceae based on concatenated sequences of all coding [file 12870_2022_3814_MOESM1_ESM.pdf]

Supporting Information Figures S1–S8, Tables S1–S4

Article title:

Comparing complete organelle genomes of holoparasitic *Christisonia kwangtungensis* with its close relatives: how different are they?

Authors:

Chi Zhang, Qianshi Lin, Jiayin Zhang, Zihao Huang, Peng Nan, Linfeng Li, Zhiping Song, Wenju Zhang, Ji Yang, and Yuguo Wang

The following supporting information is available for this article:

Figure S1. The plastid genome map of holoparasitic *C. kwangtungensis*. Genes labelled outside the outer circle are transcribed clockwise, while those inside are transcribed counterclockwise. Dashed area in the inner circle indicates the GC content of plastid genome.

Figure S2. Comparison of plastid genome structure among non-parasite

(*Lindenbergia philippensis*), hemi-parasite (*Striga asistica*), and holoparasite (*Christisonia kwangtungensis*; from top to bottom, respectively). Green lines connect homologous plastid coding genes. Green dotted lines indicate pseudogenized regions. Regions without connecting lines indicate gene loss.

Figure S3. The mitochondrial gene map of holoparasitic *C. kwangtungensis*. Genes labelled outside the outer circle are transcribed clockwise, while those inside are transcribed counterclockwise. Dashed area in the inner circle indicates the GC content of mitochondrial genome.

Figure S4. Maximum likelihood phylogeny of rpl20 in the mitochondrial genome of *C. kwangtungensis*. Phylogenetic trees show evidence of intracellular gene transfer. The clade length of trees represents the base substitution rate.

Figure S5. Gene expression in the photosynthesis pathway observed in transcriptomes of 10 species. The detected expressed genes are marked as green. Species of the same clade of *C. kwangtungensis* are in the red box. With courtesy of © www.genome.jp/kegg/kegg1.html.

Figure S6. Gene expression in porphyrin and chlorophyll metabolism pathway observed in the transcriptome of *C. kwangtungensis*. Genes with detected expression are in the red boxes. The name and number of gene expression products were marked at each node. With courtesy of © www.genome.jp/kegg/kegg1.html.

Figure S7. Mitochondrial gene content (core genes in blue and variable genes in yellow) of 21 species including *C. kwangtungensis* and A. indica. Retained genes are marked in white and lost genes are marked in black.

Figure S8. Maximum likelihood phylogenetic tree of Orobanchaceae based on concatenated sequences of all coding genes present in the plastid genome of Lindenbergia philippensis.

Table S1. Statistics of putative plastid transferred genes in mitochondrial genome of *C. kwangtungensis*. Potential donors, transfer types, fragment lengths, and bootstrap of all the putative plastid transferred genes on mitochondrial genome of *C. kwangtungensis*.

Table S2. Comparison of informative characters of the horizontally transferred

fragment *rpl20* of *C. kwangtungensis* and its related species. The alignment site with yellow background is false informative site supports *C. kwangtungensis* nesting into Lamiaceae. Absolute position refers to the position in *rpl20* of *C. kwangtungensis*.

Table S3. Mitochondrial genome sequence of Lamiales used in this study.

Table S4. Plastid genome sequence for plastid phylogeny used in this study.

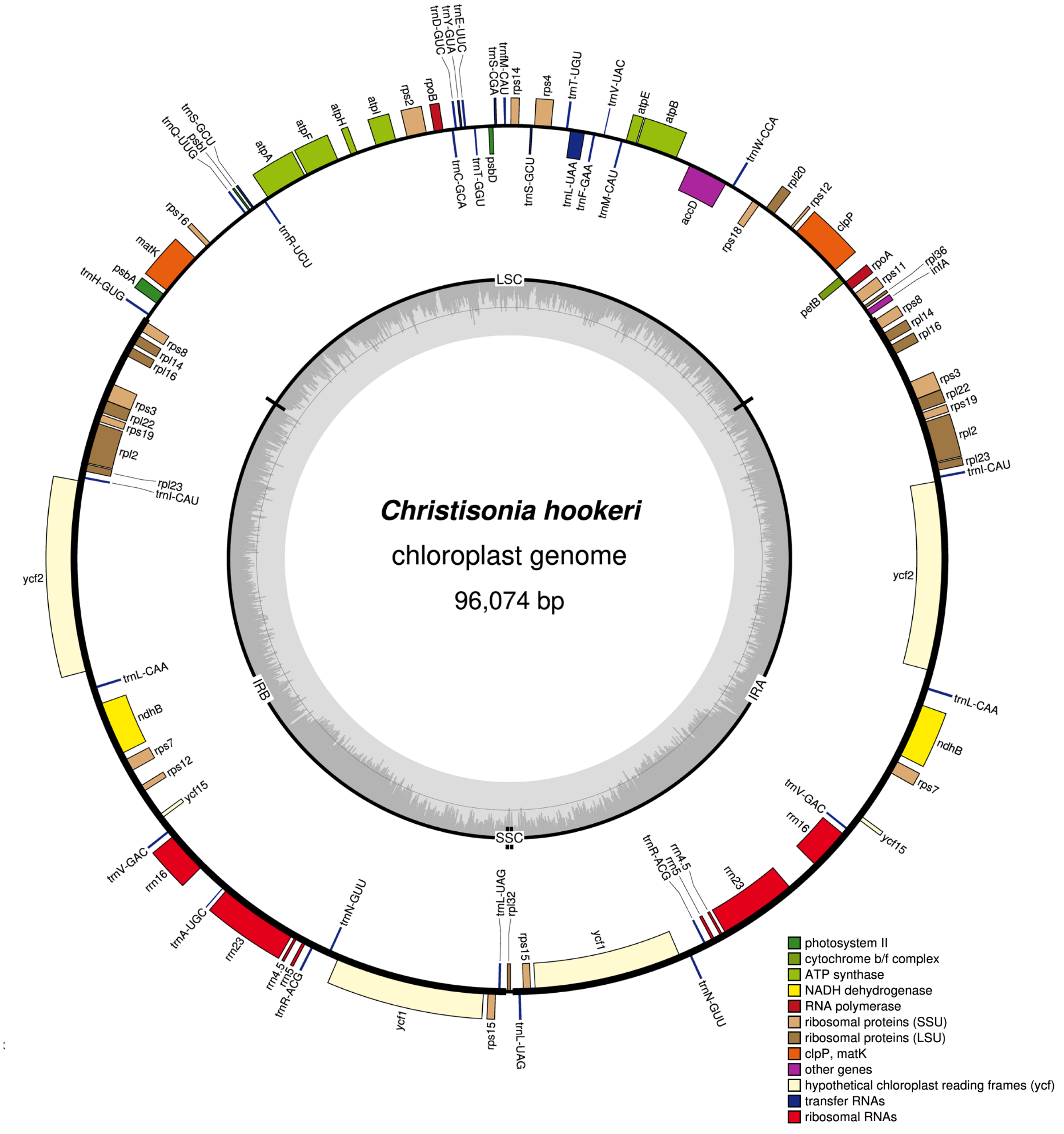
Christisonia hookeri
chloroplast genome
96,074 bp
LSC
IRA
IRB
SSC
photosystem II
cytochrome b/f complex
ATP synthase
NADH dehydrogenase
RNA polymerase
ribosomal proteins (SSU)
ribosomal proteins (LSU)
clpP, matK
other genes
hypothetical chloroplast reading frames (ycf)
transfer RNAs
ribosomal RNAs

Figure S1

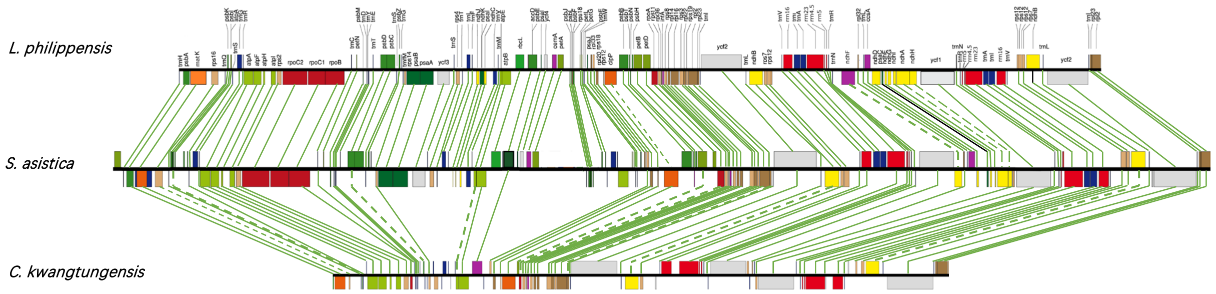

Figure S2

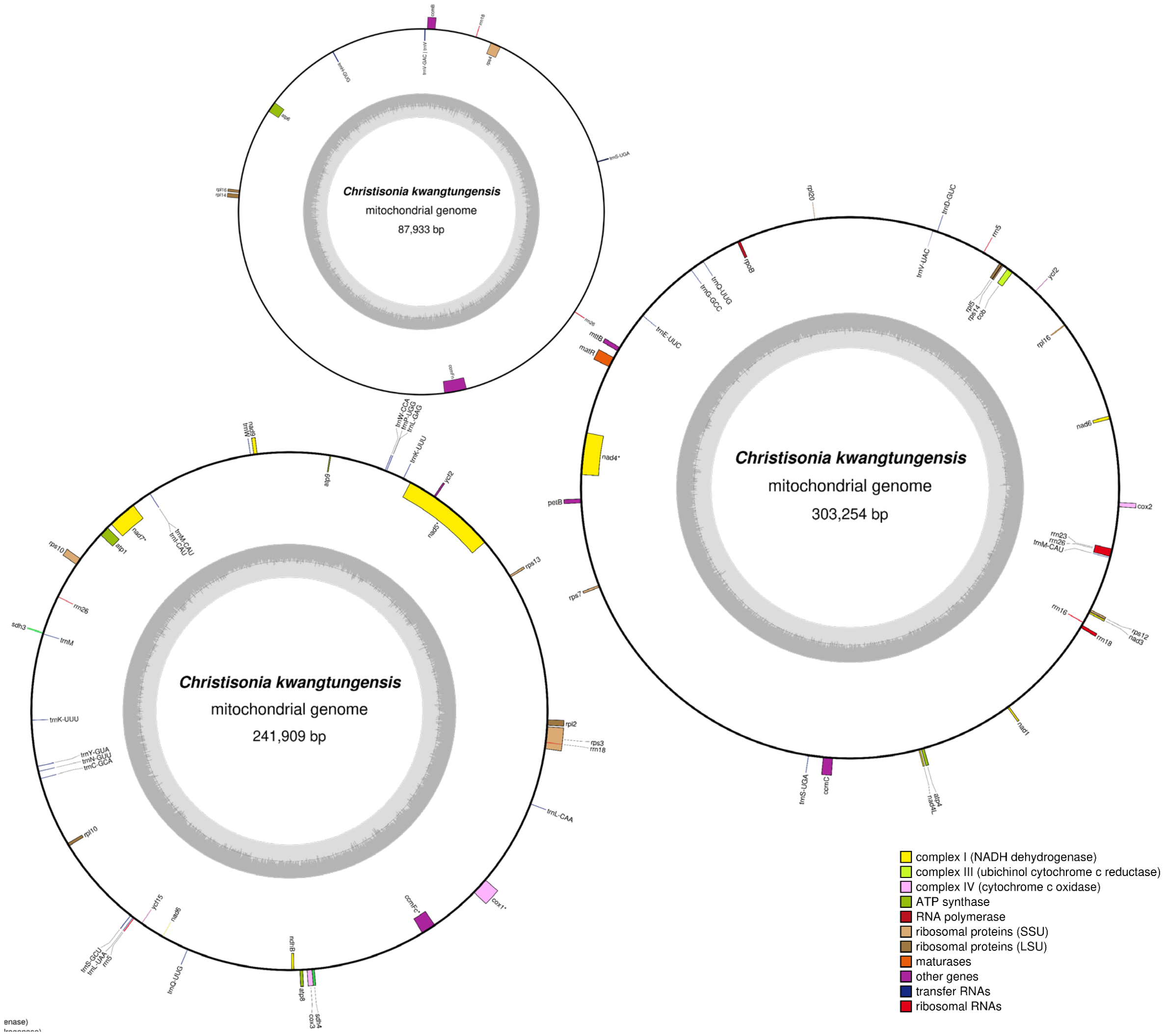
Christisonia kwangtungensis
mitochondrial genome
87,933 bp
Christisonia kwangtungensis
mitochondrial genome
303,254 bp
Christisonia kwangtungensis
mitochondrial genome
241,909 bp
complex I (NADH dehydrogenase)
complex III (ubichinol cytochrome c reductase)
complex IV (cytochrome c oxidase)
ATP synthase
RNA polymerase
ribosomal proteins (SSU)
ribosomal proteins (LSU)
maturases
other genes
transfer RNAs
ribosomal RNAs

Figure S3

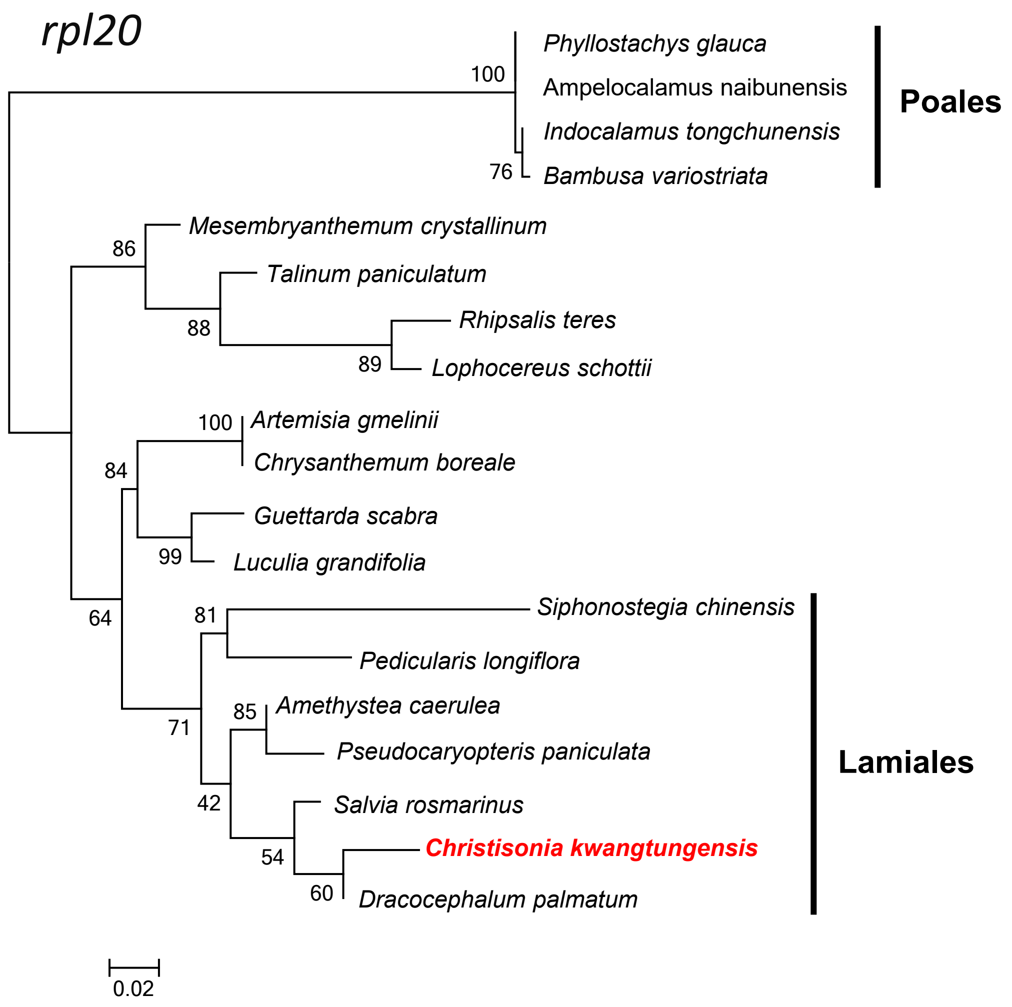
rpl20
100
Phyllostachys glauca
Ampelocalamus naibunensis
Indocalamus tongchunensis
76
Bambusa variostriata
Poales
86
Mesembryanthemum crystallinum
Talinum paniculatum
88
Rhipsalis teres
89
Lophocereus schottii
100
Artemisia gmelinii
Chrysanthemum boreale
84
Guettarda scabra
99
Luculia grandifolia
64
81
Siphonostegia chinensis
Pedicularis longiflora
85
Amethystea caerulea
71
Pseudocaryopteris paniculata
Lamiales
42
Salvia rosmarinus
54
Christisonia kwangtungensis
60
Dracocephalum palmatum
0.02

Figure S4

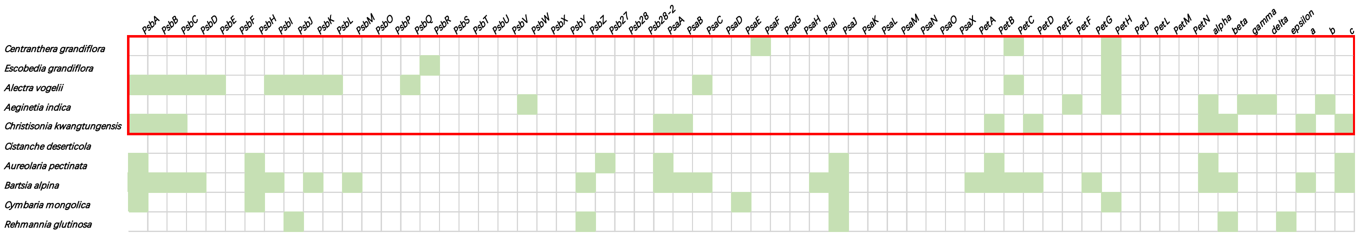
PsbA
PsbB
PsbC
PsbD
PsbE
PsbF
PsbH
PsbI
PsbJ
PsbK
PsbL
PsbM
PsbO
PsbP
PsbQ
PsbR
PsbS
PsbT
PsbU
PsbV
PsbW
PsbX
PsbY
PsbZ
Psb27
Psb28
Psb28-2
PsaA
PsaB
PsaC
PsaD
PsaE
PsaF
PsaG
PsaH
PsaI
PsaJ
PsaK
PsaL
PsaM
PsaN
PsaO
PsaX
PetA
PetB
PetC
PetD
PetE
PetF
PetG
PetH
PetJ
PetL
PetM
PetN
alpha
beta
gamma
delta
epsilon
a
b
c
Centranthera grandiflora
Escobedia grandiflora
Alectra vogelii
Aeginetia indica
Christisonia kwangtungensis
Cistanche deserticola
Aureolaria pectinata
Bartsia alpina
Cymbaria mongolica
Rehmannia glutinosa

Figure S5

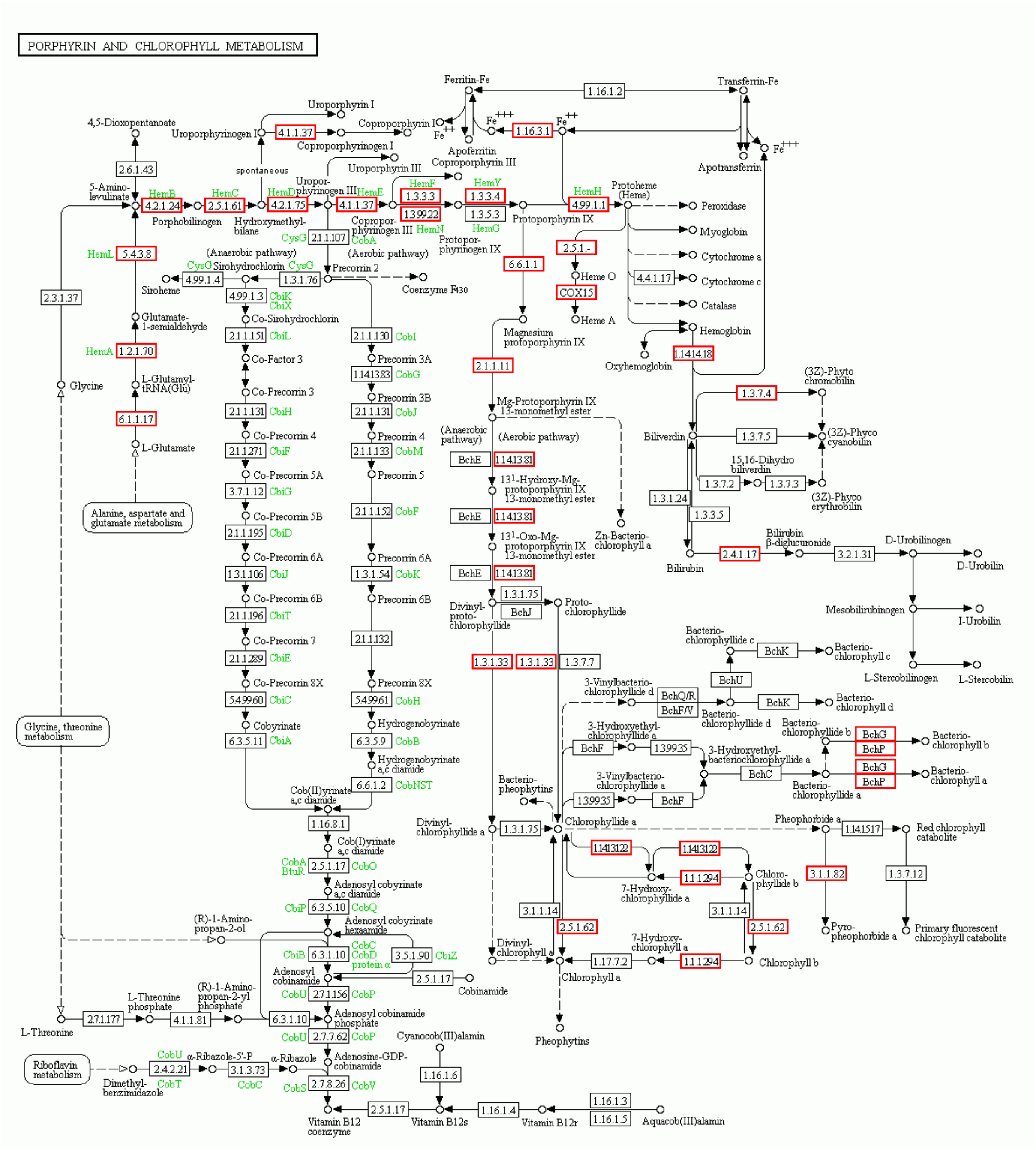

PORPHYRIN AND CHLOROPHYLL METABOLISM
Ferritin-Fe
1.16.1.2
Transferrin-Fe
Uroporphyrin I
4,5-Dioxopentanoate
Uroporphyrinogen I
4.1.1.37
Coproporphyrin I
Coproporphyrinogen I
1.16.3.1
Apoferritin
Coproporphyrin III
Apotransferrin
2.6.1.43
spontaneous
Uroporphyrin III
Uroporphyrinogen III
5-Amino-levulinate
HemB
4.2.1.24
HemC
2.5.1.61
HemD
4.2.1.75
HemE
4.1.1.37
HemF
1.3.3.3
1.3.99.22
HemY
1.3.3.4
1.3.5.3
HemH
4.99.1.1
Protoheme (Heme)
Peroxidase
Myoglobin
Cytochrome a
Cytochrome c
Catalase
Hemoglobin
Porphobilinogen
Hydroxymethyl-bilane
Coproporphyrinogen III
HemN
CobA
Protoporphyrinogen IX
HemG
Protoporphyrin IX
CysG
2.1.1.107
(Anaerobic pathway)
(Aerobic pathway)
HemL
5.4.3.8
Siroheme
4.99.1.4
Sirohydrochlorin
1.3.1.76
Precorrin 2
Coenzyme F430
6.6.1.1
2.5.1.-
Heme O
COX15
Heme A
4.4.1.17
Magnesium protoporphyrin IX
Oxyhemoglobin
1.14.14.18
2.3.1.37
Glutamate-1-semialdehyde
HemA
1.2.1.70
L-Glutamyl-tRNA(Glu)
6.1.1.17
L-Glutamate
Glycine
Alanine, aspartate and glutamate metabolism
4.99.1.3
CbiK
CbiX
Co-Sirohydrochlorin
2.1.1.151
CbiL
Co-Factor 3
Co-Precorrin 3
2.1.1.131
CbiH
Co-Precorrin 4
2.1.1.271
CbiF
Co-Precorrin 5A
3.7.1.12
CbiG
Co-Precorrin 5B
2.1.1.195
CbiD
Co-Precorrin 6A
1.3.1.106
CbiJ
Co-Precorrin 6B
2.1.1.196
CbiT
Co-Precorrin 7
2.1.1.289
CbiE
Co-Precorrin 8X
5.4.99.60
CbiC
Cobyrinate
6.3.5.11
CbiA
2.1.1.130
CobI
Precorrin 3A
1.14.13.83
CobG
Precorrin 3B
CobJ
Precorrin 4
2.1.1.133
CobM
Precorrin 5
2.1.1.152
CobF
Precorrin 6A
1.3.1.54
CobK
Precorrin 6B
2.1.1.132
Precorrin 8X
5.4.99.61
CobH
Hydrogenobyrinate
6.3.5.9
CobB
Hydrogenobyrinate a,c diamide
6.6.1.2
CobNST
Cob(II)yrinate a,c diamide
1.16.8.1
Cob(I)yrinate a,c diamide
CobA
BtuR
2.5.1.17
CobO
Adenosyl cobyrinate a,c diamide
CbiP
6.3.5.10
CobQ
Adenosyl cobyrinate hexaamide
(R)-1-Amino-propan-2-ol
CobC
CobD
CbiB
6.3.1.10
protein α
3.5.1.90
CbiZ
Adenosyl cobinamide
2.5.1.17
Cobinamide
CobU
2.7.1.156
CobP
L-Threonine phosphate
(R)-1-Amino-propan-2-yl phosphate
Glycine, threonine metabolism
L-Threonine
2.7.1.177
4.1.1.81
6.3.1.10
Adenosyl cobinamide phosphate
2.7.7.62
Cyanocob(III)alamin
Riboflavin metabolism
CobU
α-Ribazole-5'-P
α-Ribazole
Adenosine-GDP-cobinamide
2.4.2.21
3.1.3.73
Dimethyl-benzimidazole
CobT
CobC
CobS
2.7.8.26
CobV
1.16.1.6
2.5.1.17
1.16.1.4
1.16.1.3
1.16.1.5
Vitamin B12 coenzyme
Vitamin B12s
Vitamin B12r
Aquacob(III)alamin
2.1.1.11
Mg-Protoporphyrin IX 13-monomethyl ester
(Anaerobic pathway)
(Aerobic pathway)
BchE
1.14.13.81
13[1]-Hydroxy-Mg-protoporphyrin IX 13-monomethyl ester
13[1]-Oxo-Mg-protoporphyrin IX 13-monomethyl ester
Zn-Bacterio-chlorophyll a
1.3.1.75
Divinyl-proto-chlorophyllide
BchJ
Proto-chlorophyllide
1.3.1.33
1.3.7.7
3-Vinylbacterio-chlorophyllide d
BchQ/R
BchF/V
BchU
BchK
Bacterio-chlorophyllide c
Bacterio-chlorophyll c
Bacterio-chlorophyllide d
Bacterio-chlorophyll d
3-Hydroxyethyl-chlorophyllide a
BchF
1.3.99.35
3-Hydroxyethyl-bacteriochlorophyllide a
BchC
Bacterio-chlorophyllide b
BchG
BchP
Bacterio-chlorophyll b
Bacterio-chlorophyll a
Bacterio-chlorophyllide a
Bacterio-pheophytins
3-Vinylbacterio-chlorophyllide a
Divinyl-chlorophyllide a
Chlorophyllide a
Pheophorbide a
1.14.15.17
Red chlorophyll catabolite
1.14.13.122
1.1.1.294
7-Hydroxy-chlorophyllide a
Chloro-phyllide b
3.1.1.82
1.3.7.12
3.1.1.14
2.5.1.62
Pyro-pheophorbide a
Primary fluorescent chlorophyll catabolite
Divinyl-chlorophyll a
1.17.7.2
7-Hydroxy-chlorophyll a
Chlorophyll a
Chlorophyll b
Pheophytins
(3Z)-Phyto chromobilin
1.3.7.4
Biliverdin
1.3.7.5
(3Z)-Phyco cyanobilin
15,16-Dihydro biliverdin
1.3.7.2
1.3.7.3
(3Z)-Phyco erythrobilin
1.3.1.24
1.3.3.5
Bilirubin
2.4.1.17
Bilirubin β-diglucuronide
3.2.1.31
D-Urobilinogen
D-Urobilin
Mesobilirubinogen
I-Urobilin
L-Stercobilinogen
L-Stercobilin

Figure S6

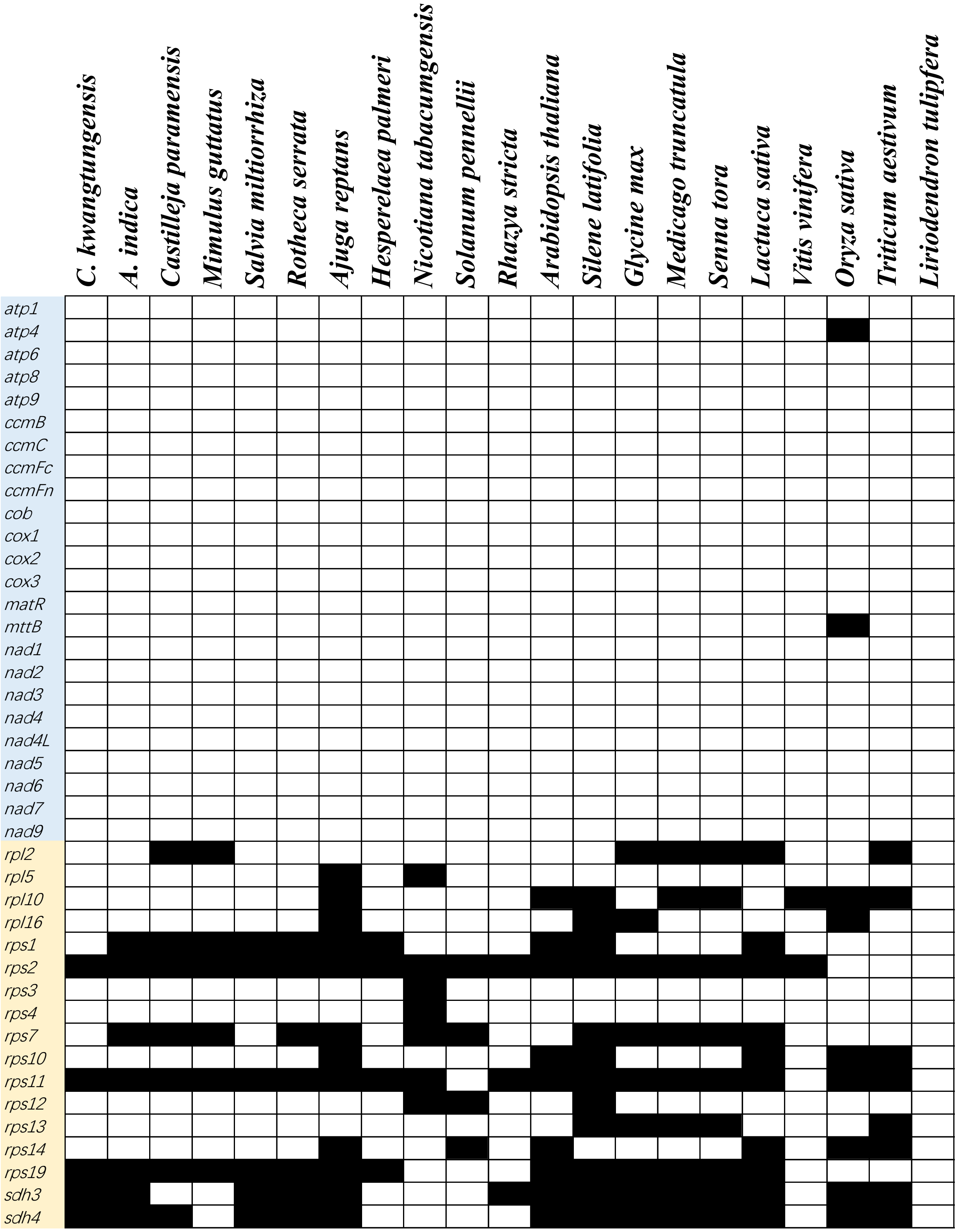
C. kwangtungensis
A. indica
Castilleja paramensis
Mimulus guttatus
Salvia miltiorrhiza
Rotheca serrata
Ajuga reptans
Hesperelaea palmeri
Nicotiana tabacumgensis
Solanum pennellii
Rhazya stricta
Arabidopsis thaliana
Silene latifolia
Glycine max
Medicago truncatula
Senna tora
Lactuca sativa
Vitis vinifera
Oryza sativa
Triticum aestivum
Liriodendron tulipfera
atp1
atp4
atp6
atp8
atp9
ccmB
ccmC
ccmFc
ccmFn
cob
cox1
cox2
cox3
matR
mttB
nad1
nad2
nad3
nad4
nad4L
nad5
nad6
nad7
nad9
rpl2
rpl5
rpl10
rpl16
rps1
rps2
rps3
rps4
rps7
rps10
rps11
rps12
rps13
rps14
rps19
sdh3
sdh4

Figure S7

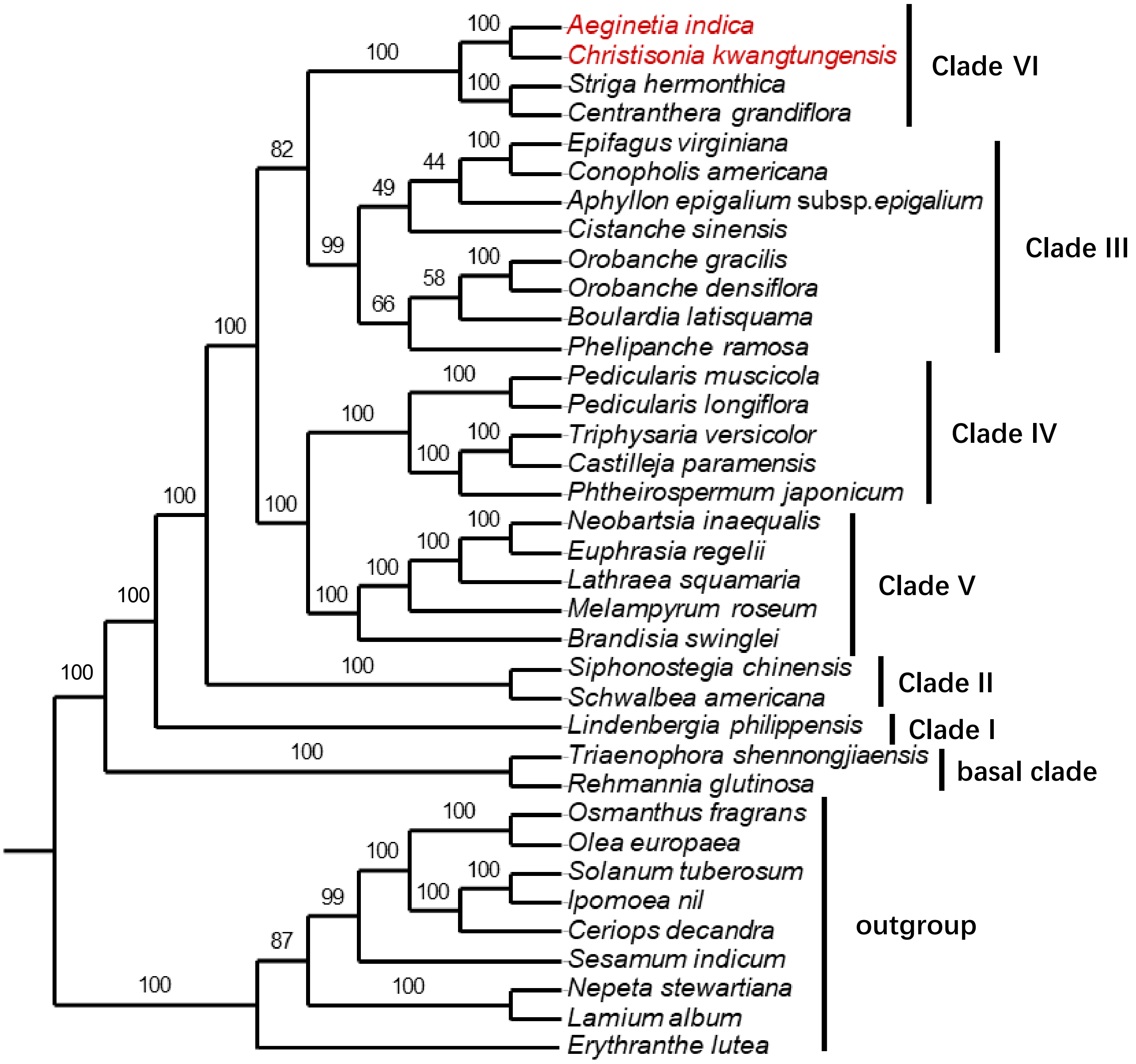

Aeginetia indica
Christisonia kwangtungensis
Striga hermonthica
Centranthera grandiflora
Epifagus virginiana
Conopholis americana
Aphyllon epigalium subsp.epigalium
Cistanche sinensis
Orobanche gracilis
Orobanche densiflora
Boulardia latisquama
Phelipanche ramosa
Pedicularis muscicola
Pedicularis longiflora
Triphysaria versicolor
Castilleja paramensis
Phtheirospermum japonicum
Neobartsia inaequalis
Euphrasia regelii
Lathraea squamaria
Melampyrum roseum
Brandisia swinglei
Siphonostegia chinensis
Schwalbea americana
Lindenbergia philippensis
Triaenophora shennongjiaensis
Rehmannia glutinosa
Osmanthus fragrans
Olea europaea
Solanum tuberosum
Ipomoea nil
Ceriops decandra
Sesamum indicum
Nepeta stewartiana
Lamium album
Erythranthe lutea
Clade VI
Clade III
Clade IV
Clade V
Clade II
Clade I
basal clade
outgroup

Figure S8

Table S1. Statistics of putative plastid transferred genes in mitochondrial genome of *C. kwangtungensis*. Potential donors, transfer types, fragment lengths, and bootstrap of all the putative plastid transferred genes on mitochondrial genome of *C. kwangtungensis*.

Gene	Potential donors	Type	Length (bp)	Bootstrap (%)
ycf2	Asparagales (Orchidaceae)	HGT	72	85
rpl16	Gentianales (Rubiaceae)	HGT	209	100
rpl14	Gentianales (Rubiaceae)	HGT	305	90
rpoB	Lamiales	IGT	570	100
ndhB	Lamiales	IGT	1130	93
rpl20	Lamiales	IGT	69	71
petB	Lamiales	IGT	576	100

Table S2. Comparison of informative characters of the horizontally transferred fragment *rpl20* of *C. kwangtungensis* and its related species. Alignment site with yellow background is false informative site supports *C. kwangtungensis* nesting into Lamiaceae. Absolute position refers to the position in *rpl20* of *C. kwangtungensis*.

Absolute position	153	156	158	164	166	175	187	192	203	211
Christisonia kwangtungensis	T	G	C	G	G	C	**A**	G	T	A
Dracocephalum palmatum	T	G	A	G	G	C	**A**	G	T	G
Salvia rosmarinus	T	G	A	G	A	C	**A**	G	T	G
Pedicularis longiflora	C	T	A	G	A	C	**G**	C	T	G
Siphonostegia chinensis	T	G	A	T	A	G	**G**	G	G	G

Table S3. Mitochondrial genome sequence of Lamiales used in this study.

Lineage	Species	Parasitic lifestyle	Data type	GenBank Accession No.
Orobanchaceae	*Castilleja paramensis*	hemi-parasitic	complete	KT959112
	Neobartsia pedicularoides	hemi-parasitic	partial	KP940485-KP940493
	Orobanche densiflora	holoparasitic	partial	KT367291-KT367292
	O. austrohispanica	holoparasitic	partial	KT366929, KT366933, KT366957-KT366958 KT366955
	O. rapum-genistae	holoparasitic	partial	KT367470, KT367476, KT367509-KT367510, KT367489
	Aphyllon epigalium	holoparasitic	partial	MH129026
	A. notocalifornicum	holoparasitic	partial	MG987142
	Aeginetia indica	holoparasitic	complete	MW851294
	Christisonia kwangtungensis	holoparasitic	complete	OM219025-OM219027
Oleaceae	*Chionanthus rupicola*	non-parasitic	complete	MG372115
	Hesperelaea palmeri	non-parasitic	complete	KX545367
	Olea cuspidata	non-parasitic	complete	MG372116
	Ol. europaea	non-parasitic	complete	MG372117
	Ol. guanchica	non-parasitic	complete	MG372120
	Ol. laperrinei	non-parasitic	complete	MG372121
Lamiaceae	*Ajuga reptans*	non-parasitic	complete	KF709392
	Salvia miltiorrhiza	non-parasitic	complete	KF177345
Gesneriaceae	*Boea hygrometrica*	non-parasitic	complete	JN107812
	Haberlea rhodopensis	non-parasitic	complete	MH757117
Phrymaceae	*Mimulus guttatus*	non-parasitic	complete	JN098455
Lentibulariaceae	*Utricularia reniformis*	non-parasitic	complete	KY774314

Table S4. Plastid genome sequence for plastid phylogeny used in this study.

Species Name	Taxonomic Groups	Life Style	GenBank Accession No.
Rehmannia glutinosa	basal clade	autotrophs	NC_034308
Triaenophora shennongjiaensis	basal clade	autotrophs	NC_039781
Lindenbergia philippensis	Clade I	autotrophs	NC_022859
Schwalbea americana	Clade II	hemi-parasites	NC_023115
Siphonostegia chinensis	Clade II	hemi-parasites	NC_046038
Aphyllon epigaliumsubsp.epigalium	Clade III	holoparasites	MH050785
Boulardia latisquama	Clade III	holoparasites	NC_025641
Cistanche sinensis	Clade III	holoparasites	MN614129
Conopholis americana	Clade III	holoparasites	NC_023131
Epifagus virginiana	Clade III	holoparasites	NC_001568
Orobanche densiflora	Clade III	holoparasites	NC_031442
Orobanche gracilis	Clade III	holoparasites	NC_023464
Phelipanche ramosa	Clade III	holoparasites	NC_023465
Triphysaria versicolor	Clade IV	hemi-parasites	NC 053793
Castilleja paramensis	Clade IV	hemi-parasites	NC_031805
Pedicularis longiflora	Clade IV	hemi-parasites	NC_046852
Pedicularis muscicola	Clade IV	hemi-parasites	NC_046853
Phtheirospermum japonicum	Clade IV	hemi-parasites	NC_053792
Brandisia swinglei	Clade V	hemi-parasites	NC_042954
Euphrasia regelii	Clade V	hemi-parasites	NC_045041
Lathraea squamaria	Clade V	holoparasites	NC_027838
Melampyrum roseum	Clade V	hemi-parasites	NC_053791
Neobartsia inaequalis	Clade V	hemi-parasites	KF922718
Aeginetia indica	Clade VI	holoparasites	MN529629
Christisonia kwangtungensis	Clade VI	holoparasites	OL362208
Centranthera grandiflora	Clade VI	hemi-parasites	NC_059747
Striga hermonthica	Clade VI	hemi-parasites	KU212372
Ceriops decandra	outgroup	autotrophs	NC_061406
Olea europaea	outgroup	autotrophs	NC_013707
Ipomoea nil	outgroup	autotrophs	NC_031159
Solanum tuberosum	outgroup	autotrophs	NC_008096
Erythranthe lutea	outgroup	autotrophs	NC_030212
Lamium album	outgroup	autotrophs	NC_036971
Nepeta stewartiana	outgroup	autotrophs	NC_057283
Osmanthus fragrans	outgroup	autotrophs	NC_042377
Sesamum indicum	outgroup	autotrophs	NC_016433
